# Exposure of Intestinal Epithelial Cells to Short- and Long-Chain Fructo-Oligosaccharides and CpG Oligodeoxynucleotides Enhances Peanut-Specific T Helper 1 Polarization

**DOI:** 10.3389/fimmu.2018.00923

**Published:** 2018-05-11

**Authors:** Simone M. Hayen, Henny G. Otten, Saskia A. Overbeek, André C. Knulst, Johan Garssen, Linette E. M. Willemsen

**Affiliations:** ^1^Department of Dermatology/Allergology, University Medical Center Utrecht, Utrecht University, Utrecht, Netherlands; ^2^Laboratory of Translational Immunology, University Medical Center Utrecht, Utrecht, Netherlands; ^3^Division of Pharmacology, Faculty of Science, Utrecht Institute for Pharmaceutical Sciences, Utrecht University, Utrecht, Netherlands; ^4^Immunology Platform, Nutricia Research, Utrecht, Netherlands

**Keywords:** allergen-specific, immunomodulation, non-digestible oligosaccharides, co-culture, epithelial cells, T cell polarization

## Abstract

**Background:**

Non-digestible oligosaccharides promote colonization of beneficial gut bacteria and have direct immunomodulatory effects. Apical exposure of intestinal epithelial cells (IECs) to short-chain galacto-oligosaccharides and long-chain fructo-oligosaccharides (scGOS/lcFOS) in a transwell co-culture model enhanced the CpG-induced (TLR-9 ligand) T helper 1 (Th1) phenotype and regulatory IL-10 response of underlying peripheral mononuclear cells (PBMCs) of healthy donors. scGOS is derived from lactose and may pose risks in severe cow’s milk allergic patients, and scFOS/lcFOS may be an alternative. The goal of this study was to determine the immunomodulatory effects of scGOS/lcFOS and scFOS/lcFOS in an allergen-specific transwell co-culture model using PBMCs from peanut-allergic patients.

**Methods:**

IECs cultured on transwell filters were apically exposed to CpG, either or not in combination with oligosaccharides. These IECs were co-cultured with basolateral PBMCs of peanut-allergic patients that were either activated with aCD3/28 or peanut extract. Basolateral cytokine production and T-cell polarization were measured and the contribution of galectin-9 and the dectin-1 receptor in immune modulation were assessed.

**Results:**

IECs exposed to CpG increased IFN-γ, IL-10, and galectin-9 production by aCD3/28-stimulated PBMCs, whereas IL-13 decreased. Both scGOS/lcFOS and scFOS/lcFOS further enhanced IFN-γ and IL-10, while suppressing IL-13 and TNF-α. In the peanut-specific model, only scFOS/lcFOS further increased IFN-γ and IL-10 production, coinciding with enhanced Th1-frequency. Expression of CRTH2 reduced after CpG exposure, and was further reduced by scFOS/lcFOS. Galectin-9 inhibitor TIM-3-Fc abrogated the additional effect of scFOS/lcFOS on peanut-specific IFN-γ production, while neutralization of the dectin-1 receptor was not effective.

**Conclusion:**

Epithelial exposure to scFOS/lcFOS enhanced the CpG-induced Th1 and regulatory IL-10 response in a peanut-specific co-culture model. These effects suggest scFOS/lcFOS as candidate for dietary adjunct in allergen-specific immunotherapy.

## Introduction

Over the past decades, the prevalence of food allergies has increased in Western countries (1, 2). Harmless food proteins are recognized as being immunogenic by the immune cells of food-allergic patients, resulting in allergic sensitization. In sen-sitized individuals, these allergens can provoke a variety of symptoms when ingested, ranging from itching and swelling in the mouth to anaphylaxis. Next to eliminating these food proteins from the diet, there are currently no therapies available for treating food allergies that induce sustained oral tolerance. Several studies were able to induce desensitization in patients undergoing oral immunotherapy (OIT), hereby increasing the eliciting dose (ED) (3–5). However, inducing sustained non-responsiveness or tolerance remains difficult and is often combined with severe side effects (1, 4). Combining OIT with additional immunomodulatory agents, such as prebiotics as dietary adjuvant, may enhance safety and efficacy of immunotherapy, and support clinical tolerance induction (6).

The gastrointestinal (GI) tract plays an important role in the development of food allergies, and is constantly discriminating between harmful and harmless antigens (7, 8). A monolayer of intestinal epithelial cells (IECs) separates the intestinal contents from the underlying immune compartment and forms a barrier, hereby keeping away harmful bacteria or antigenic proteins. They can interact with innate and adaptive immune cells *via* the release of immune mediators, such as galectin-9, or *via* cell–cell contact (9, 10). Under inflammatory conditions these IECs express pathogen recognition receptors, such as toll-like receptors (TLRs). These TLRs can recognize bacterial fragments from the gut microbiota or invading pathogens.

TLR-2 and TLR-9 have been described as important TLRs in recognition of certain probiotic strains (11). Ligation of TLR-9 by bacterial DNA rich in unmethylated CpG islands maintained intestinal homeostasis, and oral administration of a synthetic TLR-9 agonist was effective in both prevention and treatment of peanut allergy in mice by redirection of the immune response toward a T helper 1 (Th1) phenotype (12). *In vitro*, IECs apically exposed to synthetic CpG oligodeoxynucleotides (ODN) enhanced IFN-γ and IL-10 production by PBMCs in the basolateral compartment, while decreasing IL-13 (13). Therefore, targeting specific TLRs on IECs may be of interest in modulating immune responses (13).

Previous research showed that dietary intervention with specific mixtures of non-digestible oligosaccharides (prebiotics) and/or beneficial bacteria (probiotics) may help to prevent infants from developing allergic diseases (14–16). A prebiotic mixture containing short-chain galacto-oligosaccharides and long-chain fructo-oligosaccharides (scGOS/lcFOS) was able to reduce the incidence of atopic dermatitis in children at risk (15). The functioning of these prebiotics is not fully elucidated, although it is known that they can improve intestinal tolerance and promote colonization of beneficial microbiota. Indeed, children receiving such a prebiotic mixture of scGOS/lcFOS, showed an increased presence of *Bifidobacteria* and *Lactobacilli* in the gut (17). Also, the addition of scFOS or inulin to the diet increased *Bifidobac-teria* counts (18–21). Beyond their effect on the microbiome, these prebiotics may suppress mast cell and basophil degranulation by enhancing galectin-9 levels amongst others secreted by IECs (22). Furthermore, they may induce polarization of Th1 and regulatory T cells (Tregs) when combined with CpG ODN (10, 22, 23).

Previously, in a transwell co-culture model using IECs and activated PBMCs, prebiotic mixture scGOS/lcFOS indeed enhanced galectin-9 levels secreted by IECs. Apical TLR-9 ligation of IECs in the presence of scGOS/lcFOS supported the production of IFN-γ and IL-10 by PBMCs, while IL-13 production was reduced (10). Since scGOS is produced from cow’s milk-derived lactose, it may pose risks in people with severe cow’s milk allergy (24). A synbiotic mixture of scFOS/lcFOS with *Bifidobacterium breve* was also able to reduce allergic manifestations in a murine model (25). This study will compare these two mixtures and their immunomodulatory effects.

Next to galectin-9, which was shown to contribute to these immunomodulatory effects, dectin-1 may play a role in the binding of these oligosaccharides. Dectin-1 is a C-type lectin receptor that is present on human IECs and the human IEC line HT-29. It can bind carbohydrates such as β-glucans, and may therefore be a possible candidate receptor for the oligosaccharides (26, 27). Dectin-1 is expressed at high levels at entry sites for pathogens, such as the intestine, therefore, it may play an important role in immune surveillance (28).

The aim of this study was to investigate the immunomodulatory effects and mechanism of action of the two prebiotic mixtures scGOS/lcFOS and scFOS/lcFOS in a transwell co-culture model simulating the crosstalk between IECs and activated PBMCs. IECs were exposed to scGOS/lcFOS or scFOS/lcFOS in combination with CpG ODN, and co-cultured with PBMCs of peanut-allergic patients, either stimulated in an aspecific (aCD3/28) or peanut-specific manner.

## Materials and Methods

### Study Population

Fifteen peanut-allergic patients were recruited from the outpatient clinic of dermatology/allergology at the University Medical Center Utrecht. This number was calculated based on previous experiments with healthy donors. Demographic data, severity of symptoms [skin prick test (SPT) and Müller score], and the ED as established by double-blind placebo-controlled food challenge (DBPCFC) are displayed in Table 1. Inclusion criteria consisted of a type I allergic reaction to peanut, confirmed by a positive DBPCFC. Exclusion criteria were pregnancy or the continuous use of systemic immunosuppressants, such as pred-nisone. All patients gave written informed consent before enrollment in the study. Five patients that responded best to the peanut extract were asked for a second visit for additional studies. The study was reviewed and approved by the Ethics Committee of the University Medical Center Utrecht (NL51606.041.15).

**Table 1:** Patient characteristics.

Patient	Age (years)	Sex (M/F)	Müller score^b^	SPT peanut (mm)	Subjective eliciting dose (ED) (mg)	Objective ED (mg)	CAP peanut (kU/L)
N01	41	F	2	3+	10	–	1.7
N02	37	M	4	3+	0.1	300	44
N03	45	M	2	4+	100	–	1.8
N04	50	F	3	4+	10	10	12
N05	35	F	4	4+	0.1	–	85
N06^a^	27	F	2	4+	4	40	12.8
N07^a^	42	M	3	5+	Not known	300	42.7
N08	24	M	1	4+	100	>3,000	1.9
N09	24	F	3	3+	Not known	>3,000	1
N10^a^	18	F	3	4+	300	1,000	>100
N11	32	F	2	4+	10	3,000	No data
N12^a^	27	M	3	5+	0.1	1,000	66
N13	25	M	1	3+	10	–	11.2
N14^a^	26	F	2	4+	0.1	100	9.7
N15	34	F	2	4+	40	12,000	1.55

*Age, sex, Müller score, skin prick test (SPT), results of double-blind placebo-controlled food challenge (DBPCFC), and specific IgE per peanut-allergic subject*.

*^a^Subjects that visited a second time*.

*^b^Müller score 0: symptoms oral cavity, 1: symptoms of the skins and mucous membranes 2: gastro-intestinal symptoms, 3: respiratory symptoms, 4: cardiovascular symptoms*.

*SPT (mm). Diameter of 3 mm (3+) is considered positive. All patients underwent a DBPCFC, subjective, and objective effective doses are displayed*.

### PBMC Isolation

100 mL blood of peanut-allergic patients was withdrawn in heparin tubes. Blood was diluted 1:1 with 1× PBS (Sigma-Aldrich Chemie BV, the Netherlands), followed by isolation of PBMCs using a Ficoll–Paque PLUS (GE Healthcare Life Sciences, Sweden) density gradient centrifugation (2,400 rpm, 20 min). PBMCs were resuspended in RPMI 1640 (Gibco, Life Technologies, the Netherlands) with 2.5% pooled human AB serum and penicillin/streptomycin (100×, Gibco, Life Technologies).

### Culture of IECs HT-29

Undifferentiated human colon adenocarcinoma HT-29 cells (ATCC, HTB-38; passages 144–149), were cultured in 75 cm^2^ culture flasks (Greiner Bio-One B.V., the Netherlands) in McCoy’s 5 A medium (Gibco, Life Technologies, the Netherlands) supp-lemented with 10% heat-inactivated FCS (Gibco, Life Technologies, the Netherlands) and penicillin/streptomycin (100×, Gibco, Life Technologies). These cells are a representative model for crypt epithelium and can respond to bacterial stimuli (29). In the absence of an activating agent for the underlying immune cells, the HT-29 cells have very low background levels of cytokine that are being produced (13).

HT-29 cells were kept in an incubator at 37°C and 5% CO_2_. Cells were passaged once a week and medium was refreshed every 3–4 days. Previous studies have shown that HT-29 in a similar manner as polarized T84 cells contribute to the immunomodulatory effects of CpG ODN in presence or absence of oligosaccharides, and can be used as a model to mimic the cross-talk between IECs and underlying immune cells (10). Therefore, these cells were chosen for the current studies.

### IEC Transwell Co-Culture Model

**Figure 1:**
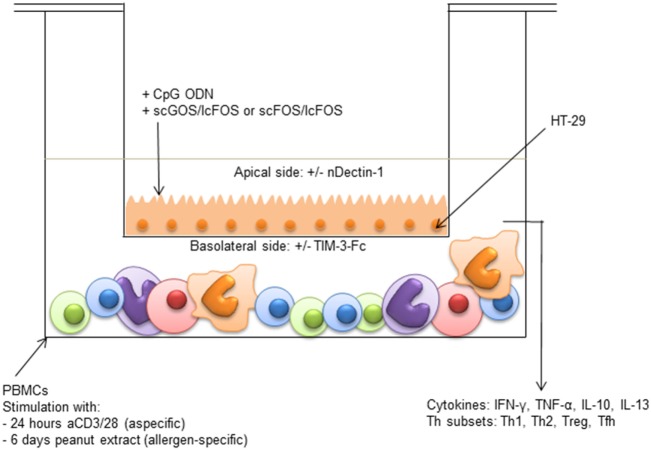
Intestinal epithelial cells (IEC) transwell co-culture model HT-29 cells (IECs) cultured in transwells were apically exposed to synthetic CpG oligodeoxynucleotides in the presence or absence of either short-chain galacto-oligosaccharides and long-chain fructo-oligosaccharides (scGOS/lcFOS) or scFOS/lcFOS. Simultaneously, 3 × 10^6^ peripheral mononuclear cells (PBMCs) in the basolateral compartment were either stimulated aspecifically for 24 h with aCD3/CD28, or for 6 days with peanut extract. Basolateral cytokine production and T cell polarization were measured. After 24 h, the insert of the aspecific model was transferred to a new plate with fresh medium, to measure production of galectin-9 by IECs. The contribution of dectin-1 and galectin-9 in the peanut-specific model was investigated by either neutralizing dectin-1 with an antibody on the apical side, or by neutralizing galectin-9 with TIM-3-Fc on the basolateral side.

One week prior to the experiment, HT-29 cells were seeded four times diluted in transwell inserts (12 well plates, 0.4 µm polyester membrane, Corning, NY, USA). After reaching confluence, IECs were apically exposed to 2.5 µM of CpG ODN (M362 ODN type C, Invitrogen) either or not combined with 0.5% w/v (5 mg/mL) of a 9:1 mixture of scGOS (Vivinal GOS syrup 45% pure, Borculo Domo, the Netherlands) and lcFOS (Raftiline HP, Orafti) or a 0.5% w/v 9:1 mixture of scFOS (Raftilose P95, Orafti) and lcFOS. In the basolateral compartment, 3 × 10^6^ PBMCs from peanut-allergic patients were either stimulated for 24 h with anti-CD3 (PeliCluster CD3, CLB-T3/4.E, 1XE) and anti-CD28 antibodies (PeliCluster CD28, CLB-CD28/1, 15E8, both 1:10,000, Sanquin, the Netherlands) or 6 days with 50 µg/mL crude peanut extract (CPE) (Figure 1). CPE was made by blending peanuts, followed by extraction with Tris/NaCL buffer (20 mM Tris, 150 mM NaCL, pH 7.2) at room temperature. After extraction, supernatant was filtered twice and diluted to the desired concentration in 1× PBS. Incubation times for the peanut-specific and aspecific model were based on previous experiences (10, 30). Due to limitation of patient material, both the aspecific and peanut-specific model could be performed once per patient.

Part of the medium was refreshed every 2–3 days. After 24 h or 6 days, culture supernatants from the basolateral compartment were stored at −20°C until cytokine measurement. After 24 h of co-culture with aCD3/28 stimulated PBMCs, the IECs were washed and the insert was transferred to a new plate with fresh medium without PBMCs for another 24 h, to determine galectin-9 production by IECs. In the peanut-specific model, galectin-9 was measured directly in the basolateral compartment after 6 days of culture.

To study the involvement of galectin-9 in immune modulation, 1 µg/mL TIM-3-Fc fusion protein (Bio-Techne, USA) was added to the basolateral compartment of the peanut-specific model, to neutralize galectin-9. Additionally, the role of dectin-1 as a candidate receptor for the oligosaccharides was investigated in the peanut-specific model, by means of a neutralizing antibody applied in the apical compartment (3 μg/mL, Bio-Techne, USA (27, 31, 32)).

### Flow Cytometric Analysis

After 24 h (aCD3/28) or 6 days (CPE), lymphocytes were collected from the basolateral compartment. Cells were stained with a panel of antibodies [CD3, CXCR3, CRTH2, CD25 (all Biolegend), CD127, FoxP3, CD4 (all eBioscience)] and CXCR5 (BD Biosciences) after which T cell polarization of Th1 (CD3^+^CD4^+^CXCR3^+^), Th2 (CD3^+^CD4^+^CRTH2^+^), Tfh (follicular T helper) (CD3^+^CD4^+^CXCR5^+^), and Treg (CD3^+^CD4^+^CD25^high^CD127^−^FoxP3^+^) was determined. FoxP3 staining was performed according to the manufacturer’s protocol (FoxP3 Transcription Factor Staining Buffer Set, Thermofisher, USA).

### Cytokine Production of PBMCs in the Basolateral Compartment

In the basolateral supernatants, IFN-γ, TNF-α, IL-10, and IL-13 and IL-4 were measured by means of ELISA, according to the manufacturer’s protocol (Ready-Set-Go, eBioscience). IL-4 was below the ELISA detection limit for both the aspecific and the allergen-specific co-culture supernatants. IL-13 and TNF-α production in the peanut-specific co-culture model was below the ELISA detection limit. In co-cultures using blood samples of four patients, PBMCs were restimulated with phorbol 12-myristate 13-acetate (PMA, 10 ng/mL, Sigma-Aldrich, the Netherlands) and ionomycin (1 µg/mL, Sigma-Aldrich, the Netherlands) for 24 h which did yield detectable levels of IL-13. Galectin-9 production was analyzed using human-galectin-9 polyclonal and biotinylated polyclonal antibodies (BioTechne). Data were analyzed by 4-parametric curve fitting using Microplate Manager software.

### Statistical Analysis

Data are expressed as mean ± SEM. The statistical significance of the data was analyzed using GraphPad Prism 7.0 software (GraphPad Software, San Diego, CA, USA). Normally distributed data were analyzed using a paired Student’s *t*-test or one-way repeated measures ANOVA followed by Bonferroni *post hoc* analysis. Not normally distributed data were first transformed (square-root or LOG) before analysis. Data were considered significant at *p* < 0.05.

## Results

### Enhanced Production of IL-10 and IFN-γ by Aspecific or Peanut-Specific Activated PBMCs Upon Combined Exposure of IECs to CpG ODN and Oligosaccharides

For this study, PBMCs of 15 peanut-allergic patients (6 male and 9 female; age 18–50; Müller 1–4) were studied in an IEC transwell co-culture model, and the immunomodulatory effects of two prebiotic mixtures were assessed. Hereto, PBMCs of these peanut-allergic patients were either stimulated aspecifically with aCD3/28 or peanut-specific by using a crude peanut extract. These PBMCs were co-cultured with IECs that were apically exposed to prebiotic mixtures in the presence of CpG ODN (TLR-9 ligand).

**Figure 2:**
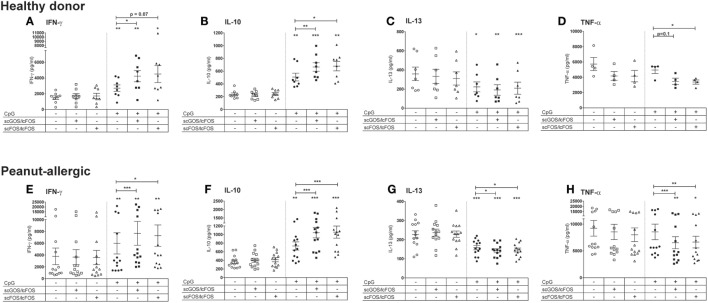
Comparison aspecific co-culture model between healthy donors and peanut-allergic donors. Apical exposure of intestinal epithelial cells (IECs) to non-digestible oligosaccharides in absence of CpG oligodeoxynucleotides (ODN) did not affect cytokine concentrations in the basolateral compartment **(A–H)**. CpG exposure increased IFN-γ and IL-10 production **(A,B)** in healthy donors, which was further increased by the combined exposure to CpG ODN and oligosaccharides. In addition, IL-13 was decreased by CpG exposure alone **(C)** and TNF-α production decreased in the combined presence of oligosaccharides and CpG **(D)**. Peanut-allergic donors showed similar results in terms of these response patterns upon exposure of IECs to CpG, oligosaccharides, or the combination of CpG and oligosaccharides **(E–H)**.

**Figure 3:**
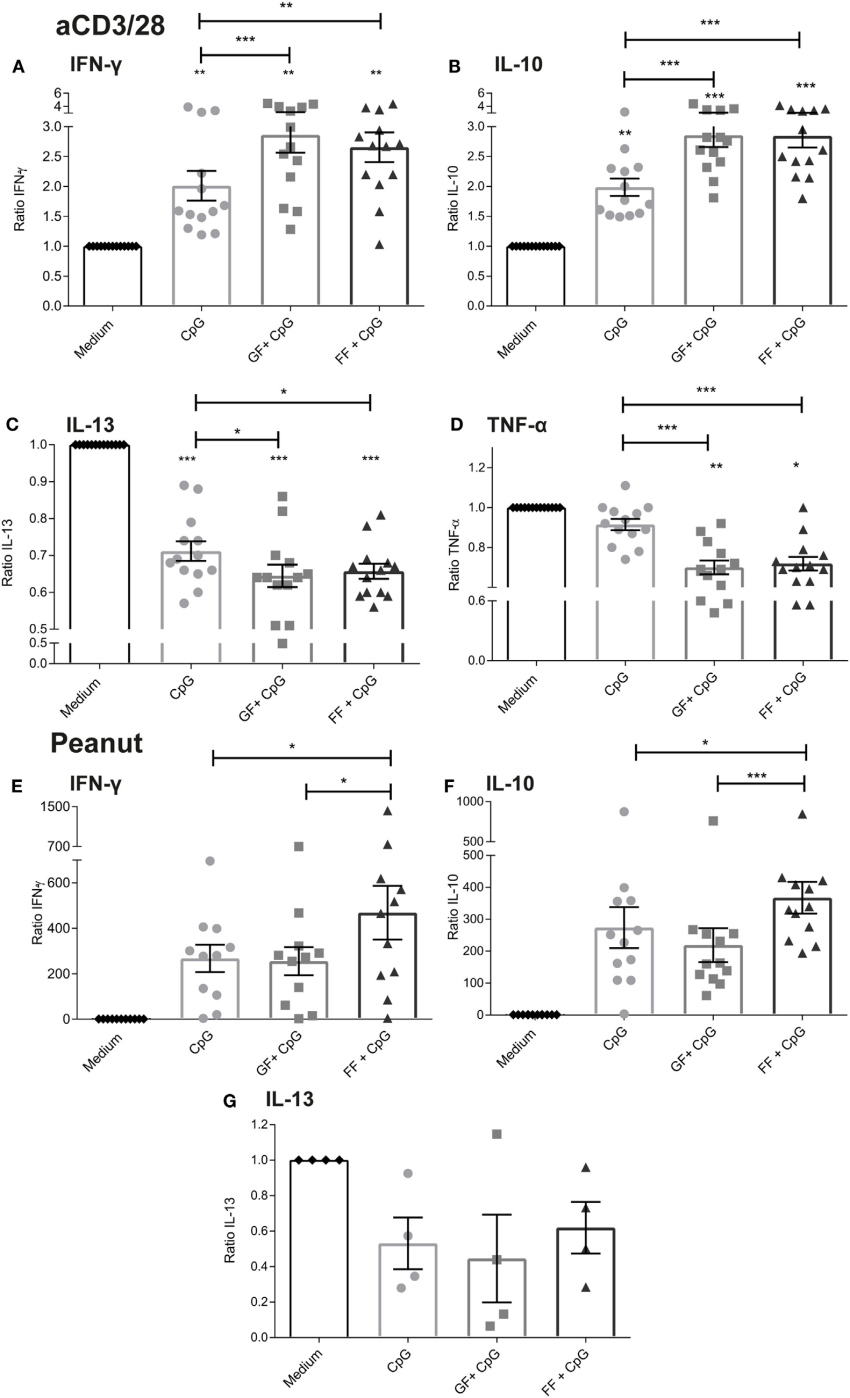
Enhanced production of IL-10 and IFN-γ by aspecific or peanut-specific activated peripheral mononuclear cells upon combined exposure of intestinal epithelial cells (IECs) to CpG oligodeoxynucleotides (ODN) and oligosaccharides. Exposure of IECs to CpG ODN in combination with short-chain galacto-oligosaccharides and long-chain fructo-oligosaccharides (scGOS/lcFOS) (GF) or scFOS/lcFOS (FF) enhanced basolateral IFN-γ and IL-10 production in the aspecific model **(A,B)**. IL-13 production was decreased in the aspecific model upon exposure of IECs to CpG ODN and was further decreased by oligosaccharides **(C)**. TNF-α production was decreased in the combined presence of scGOS/lcFOS and scFOS/lcFOS **(D)**. Only scFOS/lcFOS was able to enhance basolateral IFN-γ and IL-10 production induced by CpG ODN in the peanut-specific model **(E,F)**. IL-13 was measured after restimulation in four peanut-allergic patients **(G)**. Data are represented as ratio’s compared to the medium control and represent *n* = 12–13 peanut-allergic patients, mean ± SEM, *P < 0.05, **P < 0.01, ***P < 0.001 by one-way ANOVA.

The peanut-allergic patients showed similar responses in this aspecific model as healthy donors (Figure 2). Apical exposure of IECs to oligosaccharides alone did not affect cytokine concentra-tions in the basolateral compartment, but modified CpG ODN-induced immune responses in the aspecific co-culture model (Figure 2). To better appreciate these effects, the subsequent data of the CpG exposed IECs co-cultured with PBMCs of peanut-allergic patients are represented as ratio’s compared to the intrinsic medium control (Figure 3). Exposure of IECs to CpG ODN resulted in increased basolateral IFN-γ and IL-10 release by PBMCs of both healthy and allergic donors in the aspecific co-culture model (Figures 2A,E,F and 3A,B). Both scGOS/lcFOS and scFOS/lcFOS further significantly enhanced this CpG induced increase in IFN-γ and IL-10 in the aspecific co-culture model. In addition, IL-13 production was decreased by CpG ODN, and was further significantly decreased in the presence of the oligosaccharides in peanut-allergic patients (Figures 2C,G and 3C). Combined exposure of IECs to CpG ODN and scGOS/lcFOS or scFOS/lcFOS also resulted in a significant decrease in TNF-α, while CpG alone did not (Figures 2D,H and 3D). In previous studies, in absence of epithelial cells the CpG ODN did enhance IL-10 and reduced IL-13 secretion by activated PBMCs, but was unable to further enhance IFN-γ production compared to the control sample. Only in the presence of HT-29 cells CpG ODN increased IFN-γ production of underlying immune cells and additional exposure to oligosaccharides further increased this (10, 13).

In the peanut-specific co-culture model, only scFOS/lcFOS was able to further significantly enhance the CpG mediated increase in basolateral IFN-γ and IL-10 production (Figures 3E,F). In the peanut-specific model, IL-13 was only detectable after restimulation of the cells with PMA and ionomycin for 24 h, and shows a similar pattern as in the aspecific model (analyzed for *n* = 4 donors, Figure 3G).

**Figure 4:**
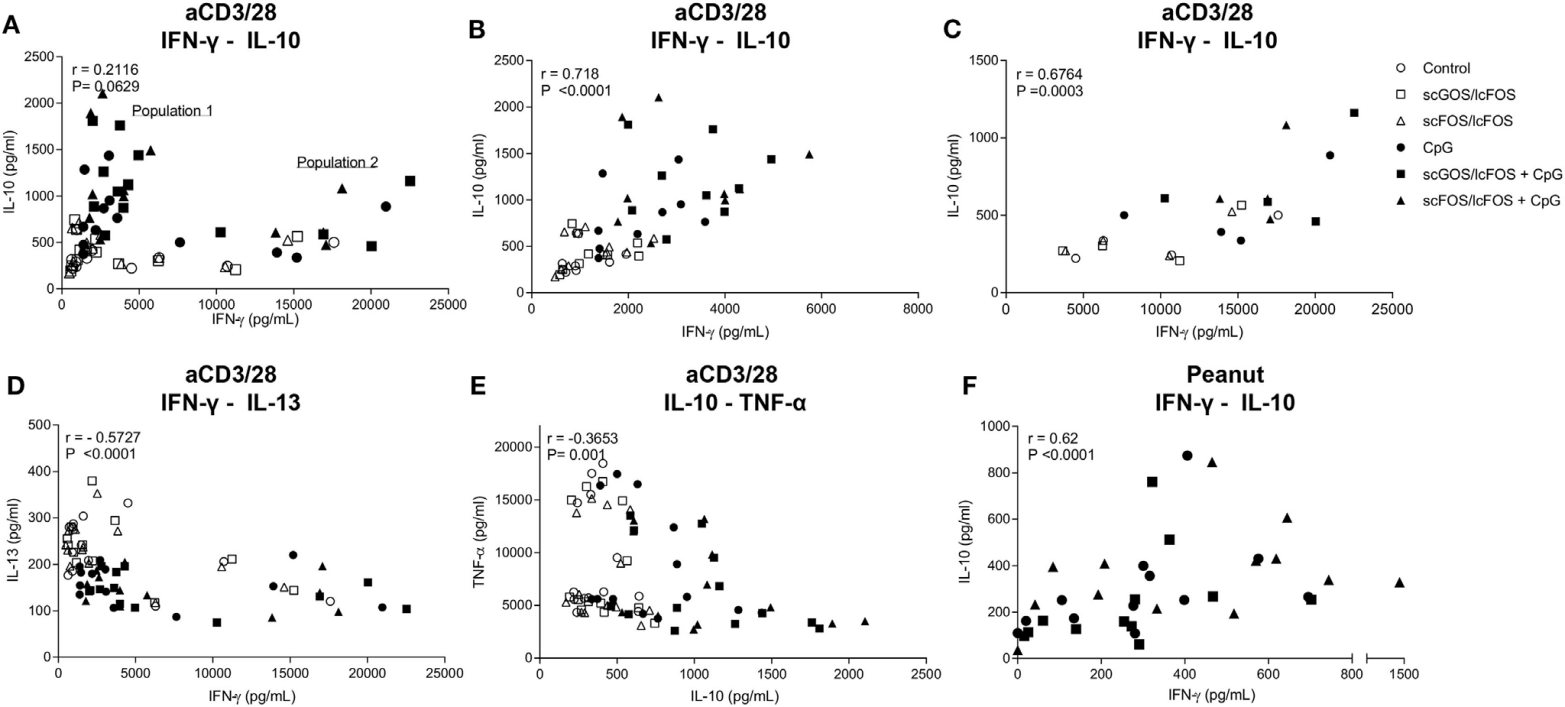
Correlation of cytokine production. IFN-γ and IL-10 concentrations were positively correlated in both the aspecific and peanut-specific co-culture models **(A–C,F)**. In the aspecific model, a clear distinction could be made into two populations **(B,C)**. Population 2 was comprised of four patients for all data points. A negative correlation existed between IFN-γ and IL-13 concentration **(D)** and TNF-α and IL-10 concentration **(E)**. Data represent *n* = 12–13 peanut-allergic patients. Correlation was tested with Spearman’s rank correlation coefficient.

IFN-γ and IL-10 concentrations were positively correlated in both the aspecific and peanut-specific co-culture models (Figures 4A–C,F). In the aspecific co-culture model, two distinct populations were observed; population 2 consisted of four patients (N04, N06, N08, and N14) for all epithelial stimuli. This indicated that patients can respond differently in terms of cytokine production pattern, however, this was not related to the demographic data from Table 1. In addition, in both populations this positive correlation was observed. In the aspecific model, IFN-γ and IL-13 (Figure 4D) and TNF-α and IL-10 concentrations (Figure 4E) were negatively correlated.

### Increased Galectin-9 Production by IECs Upon Apical Exposure to CpG ODN in Presence or Absence of Oligosaccharides

**Figure 5:**
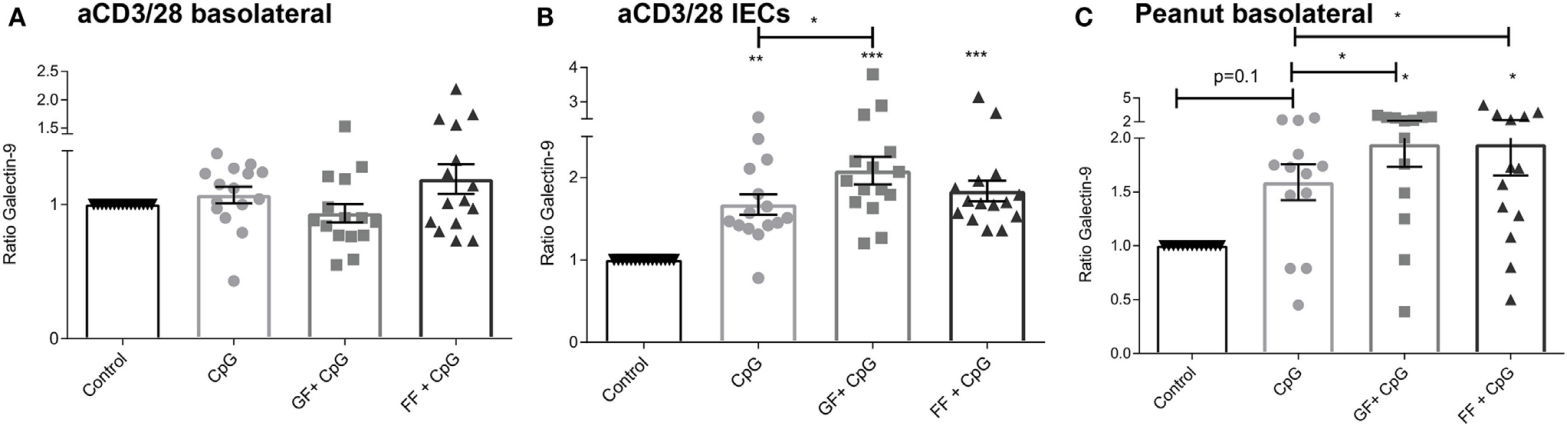
Increased galectin-9 production by intestinal epithelial cells (IECs) upon apical exposure to CpG oligodeoxynucleotides in the presence or absence of oligosaccharides. In the aspecific co-culture model, no differences in basolateral galectin-9 were observed after 24 h **(A)**. Exposure of IECs to oligosaccharides alone did not influence galectin-9 levels, while CpG exposure influenced galectin-9 release by IECs after 48 h, which was further enhanced by short-chain galacto-oligosaccharides and long-chain fructo-oligosaccharides (scGOS/lcFOS) (GF), but not by scFOS/lcFOS (FF) **(B)**. In the peanut-specific model, both oligosaccharide mixtures further enhanced galectin-9 concentrations **(C)**. Data represent *n* = 15 peanut-allergic patients, mean ± SEM, *P < 0.05, **P < 0.01, ***P < 0.001 by one-way ANOVA.

Galectins are soluble-type lectins that have a binding specificity for β-galactoside sugars. Galectins among others are expressed and secreted by IECs, and contribute to immunomodulatory functions. Total galectin-9 concentrations were measured in the basolateral compartment after 24 h (aspecific model) and 6 days (peanut-specific model) (Figures 5A,C). Also, IEC-released galectin-9 was measured in the aspecific co-culture model (Figure 5B). Since the IECs in the peanut-specific model were already cultured for 6 days, we did not measure galectin-9 levels of these IECs separately.

Exposure of IECs to oligosaccharides alone did not influence galectin-9 concentrations, and data are shown as ratio of the intrinsic medium control. No difference in basolateral galectin-9 concentration was observed after 24 h in the aspecific co-culture (Figure 5A), while IECs after another 24 h of culture without PBMCs showed an increased galectin-9 production when exposed to CpG ODN (Figure 5B). This was further significantly enhanced by combined exposure of IECs to both CpG and scGOS/lcFOS. In the peanut-specific co-culture model, combined exposure to CpG and both oligosaccharide mixtures significantly enhanced galectin-9, while CpG exposure alone showed a similar tendency.

### Increased Percentage of Treg and Tfh Subsets in the Peanut-Specific Co-Culture Model Upon Exposure of IECs to CpG ODN

**Figure 6:**
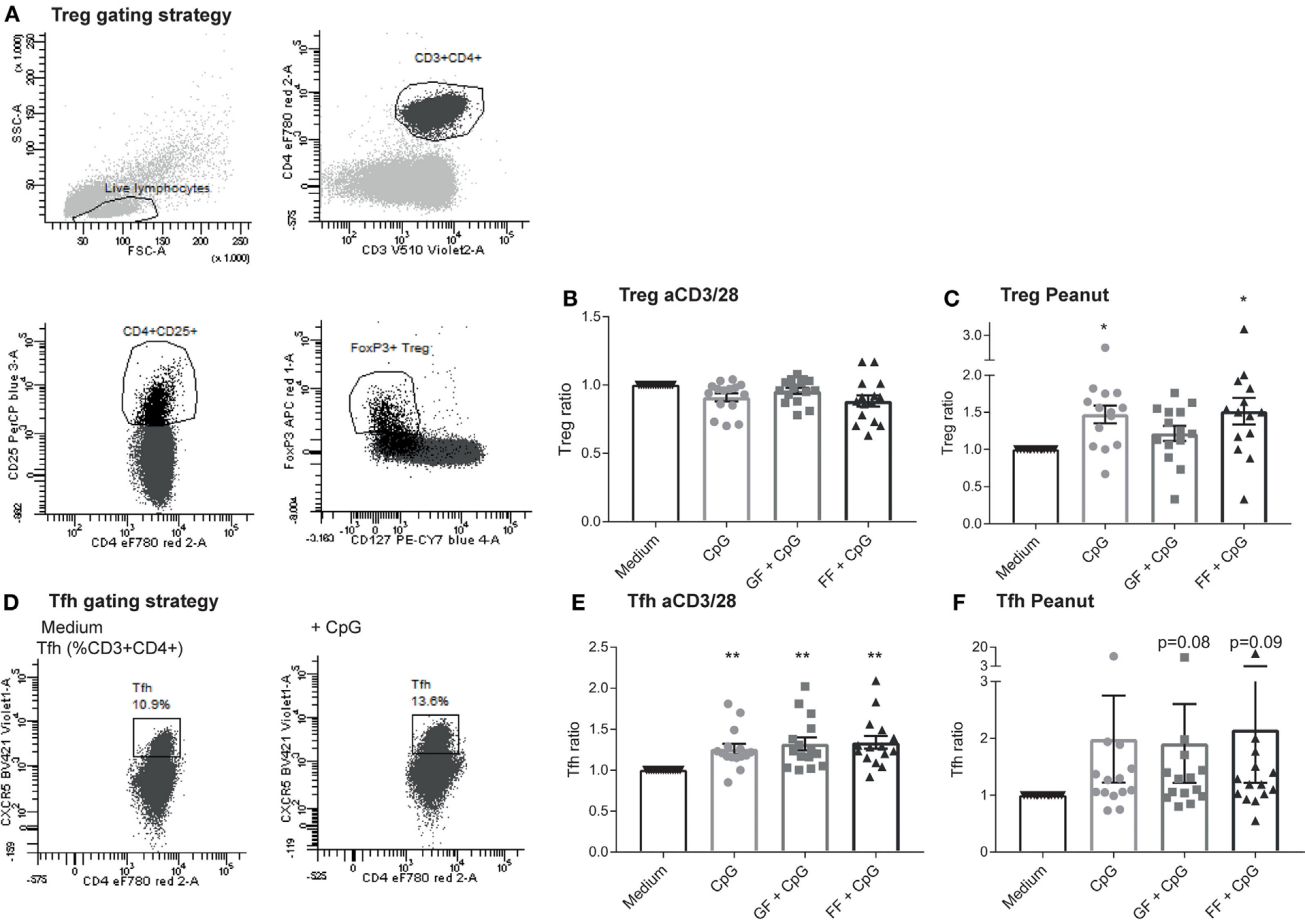
Increased percentage of regulatory T cells (Treg) and Tfh subsets in the peanut-specific co-culture model upon exposure to intestinal epithelial cells (IECs) to CpG oligodeoxynucleotides (ODN). Tregs were gated as indicated **(A)**. Treg polarization in the aspecific co-culture model was not altered upon exposure of IECs to CpG ODN, and no contributions of the oligosaccharides were observed **(B)**, whereas in the peanut-specific model, the Treg population increased upon CpG exposure **(C)**. Tfh were gated as indicated **(D)**. The percentage of Tfh cells increased upon CpG exposure of IECs, but was not further enhanced by the oligosaccharides **(E)**. In the peanut-specific model, Tfh also increased upon CpG exposure of IECs **(F)**. Data represent *n* = 15 peanut-allergic patients, mean ± SEM, *P < 0.05, **P < 0.01, ***P < 0.001 by one-way ANOVA. Short-chain galacto-oligosaccharides and long-chain fructo-oligosaccharides (scGOS/lcFOS) (GF), scFOS/lcFOS (FF).

Allergy is caused by a combination of overactivation of Th2 cells and impaired active suppression mediated by Treg and regulatory cytokines or anergy induction (33). Therefore, T cell polarization was assessed to determine whether this could be affected by the oligosaccharide mixtures. The Treg population (CD4^+^CD25^high^CD127^−^FoxP3^+^, Figure 6A) remained stable in the aspecific co-culture model (Figure 6B), while it significantly increased in the peanut-specific model upon exposure of IECs to CpG ODN (Figure 6C). In addition, the Tfh subset (Figure 6D) in the aspecific model was significantly increased (Figure 6E), and a similar trend in the peanut-specific model was observed (Figure 6F). Tfh can produce IL-21, which can inhibit class switching to IgE (34). Intracellular IL-21 was measured in the aspecific co-culture model after restimulation with PMA and ionomycin, and was increased after CpG exposure in presence or absence of oligosaccharides (data not shown).

### Increased Th1 Subset in a Peanut-Specific Co-Culture Model Upon Exposure of IECs to CpG, While CRTH2 Is Downregulated

**Figure 7:**
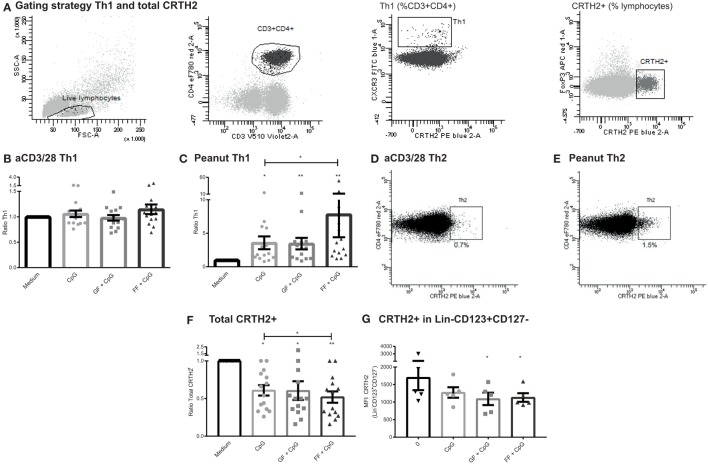
Increased T helper 1 (Th1) subset in a peanut-specific co-culture model upon exposure of intestinal epithelial cells (IECs) to CpG, while CRTH2 is downregulated. Gating strategy of Th1 and CRTH2^+^ cells **(A)**. The Th1 subset of the aspecific co-culture model was not affected after CpG exposure of IECs **(B)**. In the peanut-specific model, IECs exposed to CpG oligodeoxynucleotides (ODN) mediated an increase in the basolateral Th1 population, which was further enhanced by short- and long-chain fructo-oligosaccharides (scFOS/lcFOS) (FF) **(C)**. A representative patient shows that the percentage of Th2 cells was higher in the peanut-specific model **(D,E)**. A significant decrease in surface marker CRTH2 in the peanut-specific peripheral mononuclear cells fraction was observed, after apical exposure of IECs to CpG ODN, which was further decreased by scFOS/lcFOS **(F)**. Reduction of Lin^−^CD123^+^CD127^−^ coincided with a reduction in CRTH2 expression in this cell subset **(G)**. *N* = 15 peanut-allergic patients, mean ± SEM, *P < 0.05, **P < 0.01, ***P < 0.001 by one-way ANOVA, scGOS/lcFOS (GF).

Similar to the Treg population, no changes were observed in the Th1 or Th2 subset (gating Figure 7A) in the different IEC exposure conditions of the aspecific co-culture model (Figure 7B, Th2 data not shown). However, in the peanut-specific model, IECs exposed to CpG ODN in the apical compartment enhanced the percentage of basolateral Th1 cells (Figure 7C). This Th1 polarization was further significantly enhanced when IECs were exposed to both CpG ODN and scFOS/lcFOS, but not with scGOS/lcFOS.

The Th1 subset comprised a significantly lower percentage in peanut-specific stimulated PBMCs compared to aspecifically activated PBMCs; by contrast the Th2 subset was increased up to twofold (Figures 7D,E). This indicates that the stimulation with the peanut extract induced a peanut-specific Th2 response. Although this response is higher in the peanut-specific model, no changes were observed in percentages of Th2 cells in the separate conditions of the peanut- or aspecific co-culture model (data not shown). However, the CRTH2 expression significantly decreased in the peanut-specific PBMC fraction when IECs were apically exposed to CpG ODN (Figure 7F). This was further significantly decreased by scFOS/lcFOS. CRTH2 is a prostaglandin D2 receptor, and is a surface marker that is selectively expressed on, for instance, Th2 cells, but also on other cells involved in allergy, such as basophils and eosinophils (35, 36). This reduction in CRTH2 corresponded with a decrease in a recently described new subset, a Lin^−^CD123^+^CD127^low^ population (Figure 7G) which shares some markers with both basophils and ILCs (37).

### Neutralization of Galectin-9 Abrogates IFN-γ Production in the Peanut-Specific Co-Culture Model

**Figure 8:**
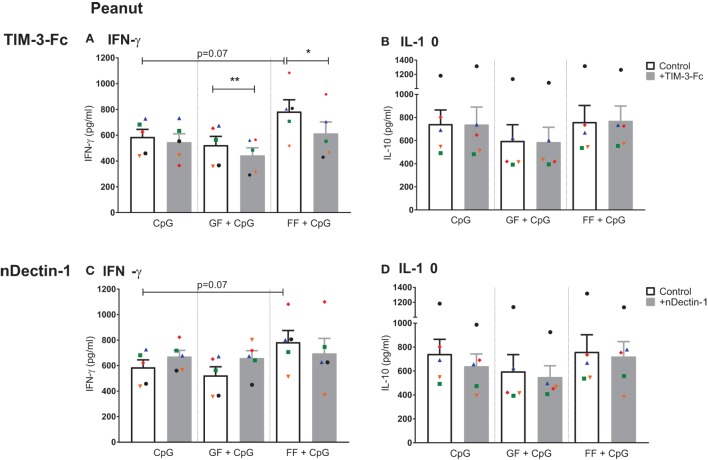
Neutralization of galectin-9 abrogates IFN-γ production in the peanut-specific co-culture model, while neutralization of the dectin-1 receptor does not affect IFN-γ and IL-10 production. Addition of TIM-3-Fc to the peanut-specific co-culture abrogated additional IFN-γ production by short- and long-chain fructo-oligosaccharides (scFOS/lcFOS) (FF), and also decreased IFN-γ production upon combined exposure to CpG oligodeoxynucleotides and scGOS/lcFOS (GF) **(A)**. IL-10 production was not influenced by addition of TIM-3-Fc **(B)**. Neutralization of dectin-1 receptor on HT-29 cells did not affect IFN-γ or IL-10 production **(C,D)**. Data represent *n* = 5 peanut-allergic patients, mean ± SEM, *P < 0.05, **P < 0.01, ***P < 0.001 by one-way ANOVA or Student’s paired *t*-test.

Previous research in our group indicated that the neutralization of galectin-9 by TIM-3-Fc in an aspecific co-culture model with CpG ODN and scGOS/lcFOS abrogated the increase in IFN-γ and IL-10 production by PBMCs (10). To examine the contribution of galectin-9 in the immunomodulatory effects of scFOS/lcFOS in the peanut-specific co-culture model, basolateral galectin-9 was inhibited by TIM-3-Fc. In these donors, scFOS/lcFOS tended to increase IFN-γ further than CpG ODN alone, this effect was abrogated with TIM-3-Fc (Figure 8A). This indicates that galectin-9 is involved in the scFOS/lcFOS induced increase of IFN-γ when added together with CpG ODN in the peanut-specific co-culture model. Although combined exposure to both scGOS/lcFOS and CpG ODN did not further increase the IFN-γ concentration compared to CpG ODN, neutralization of galectin-9 by TIM-3-Fc reduced IFN-γ production, hereby also indicating the involvement of galectin-9 in the IFN-γ response in presence of scGOS/lcFOS. IL-10 concentrations were not further increased by scGOS/lcFOS or scFOS/lcFOS, and also no effects of TIM-3-Fc were observed (Figure 8B).

### Neutralization of the Dectin-1 Receptor Does Not Affect IFN-γ and IL-10 Production in the Peanut-Specific Co-Culture in Which IECs Are Exposed to Both CpG and Oligosaccharides

Since dectin-1 is a C-type lectin receptor and can bind carbohydrates, it may be a possible candidate receptor for oligosaccharides to exert their functions. Neutralization of the dectin-1 receptor (nDectin) showed varying results in the patient samples. IFN-γ and IL-10 production were not affected by neutralization of the dectin-1 receptor on HT-29 cells (Figures 8C,D).

## Discussion

This research explored and compared the immunomodulatory capacities of oligosaccharide mixtures scGOS/lcFOS and scFOS/lcFOS to gain insight in the underlying mechanisms of the observed allergy-reducing effects. To our knowledge, the immunomodulatory capacities of scFOS/lcFOS in *in vitro* models have not been investigated previously. In addition, this study was performed with cells of peanut-allergic patients instead of healthy donors. In the aspecific co-culture model with PBMCs of peanut-allergic patients, both oligosaccharide mixtures were effective in significantly enhancing IFN-γ and IL-10, while decreasing IL-13 and TNF-α production. By contrast, in presence of TLR-9 ligation with CpG ODN, the combination with scFOS/lcFOS rather than scGOS/lcFOS was effective in enhancing this Th1 and regulatory IL-10 response in a peanut-specific model. This correlated increase in both IFN-γ and IL-10 production was described previously (13), and depended on the presence of the IECs in the co-culture model. These IECs can modulate immune responses, and under the influence of TLR9 ligand CpG ODN, both IFN-γ and IL-10 were upregulated and this was further enhanced by oligosaccharides. Although the IL-13 production in the peanut-specific model could only be determined in a small sample size, it showed a similar trend as the aspecific model. In addition, a significant decrease in prostaglandin receptor CRTH2 expression was observed in the peanut-specific model when IECs were exposed to both scFOS/lcFOS and CpG ODN. This receptor is associated with allergy and inflammation, since activation of this receptor can induce chemotaxis of Th2 cells, eosinophils, or basophils to sites of inflammation (38–40). Therefore, we can conclude that the overall cytokine balance of the observed effector response of CpG ODN combined with scFOS/lcFOS in a peanut-specific model is favored toward a Th1 and regulatory IL-10 response, driving away from the inflammatory allergic phenotype. The latter is supported by the observed decrease of TNF-α and the negative correlation between IL-10 and TNF-α.

In the aspecific model of both healthy as well as peanut-allergic donors, scGOS/lcFOS and scFOS/lcFOS significantly enhanced the effect of CpG ODN. Typically in the peanut-specific model, only scFOS/lcFOS was capable of enhancing the regulatory Th1 response when combined with CpG ODN in terms of increased IFN-γ and IL-10 production and Th1 polarization. This may be related to structural differences between these oligosaccharides. scGOS is synthesized from lactose by β-galactosidase, and consists of galactose polymers in combination with a glucose moiety on the reducing terminus, with a degree of polymerization (DP) of less than 10 monomers (41). In contrast, scFOS is derived from inulin, and consists of fructose polymers with a DP of 2–6 (41). Currently, it is not known why scGOS/lcFOS did not enhance CpG effects in the peanut-specific model; however, this may be related to the allergen-specific way of stimulation of the PBMCs. These differences in stimulation indicate the importance of confirming the effects in an allergen-specific model beyond the use of aspecific stimulation models. The differences between cytokine responses of scGOS/lcFOS and scFOS/lcFOS in this transwell co-culture model could be evaluated more in depth with a concentration-response study, however, due to the limited amount of PBMCs obtained from peanut-allergic patients, this was not possible in this study.

Although additional cytokine effects of the prebiotic mixtures in combination with CpG were observed in the aspecific co-culture model, these additional effects could not be directly linked to the Th1 cell polarization which was previously shown using PBMCs derived from healthy donor buffy coats (22). However, in the peanut-specific model the additional effect of scFOS/lcFOS on top of the CpG ODN effect on IFN-γ production could be linked to increased Th1 percentages. An explanation for missing this direct link between the additional cytokine production by the oligosaccharides and T cell polarization is that cytokines, IFN-γ and IL-10, can be produced by other cell subsets than Th1 cells or Tregs within the PBMCs. For instance, NK cells, CTLs and ILCs can produce IFN-γ (42), whereas monocytes and B-cells can also produce IL-10 (13, 43). The decrease in IL-13 was not associated with a reduction in the Th2 subset, but may be explained by the decrease in the total CRTH2 population, or the increase of IFN-γ. This cytokine is known to be able to inhibit Th2-type responses (44). It could be possible that the non-digestible oligosaccharides exert their functions not only on T cell level, but also influence other cells in the co-culture model, which should be further investigated. Cytokine production can also be influenced by age. This study depended on patients that voluntarily donated blood; therefore, the age of patients was not homogeneous. However, no correlations between age and cytokine production were observed (data not shown). The choice for using HT-29 cells in this study was based on previous research. For the future, it would be interesting to validate these results with for instance primary epithelial material from (allergic) patients.

In the peanut-specific model, an additional increase in basolateral galectin-9 concentration was observed when IECs were exposed to the combination of oligosaccharides and CpG ODN. This coincided with a decrease in IFN-γ production in the peanut-specific co-culture model when galectin-9 was neutralized by TIM-3-Fc. Therefore, we assume that also in an allergen-specific setting, galectin-9 may mediate the immunomodulatory effect in the case of scFOS/lcFOS, as was described previously (10). Next to the role of galectin-9, we assessed whether oligosaccharide mixtures exert their functions *via* C-type lectin receptor dectin-1 which is present on human IECs and HT-29 cells (27). IFN-γ production was not significantly affected after neutralization of this receptor, indicating that dectin-1 might not be important in the recognition of non-digestible oligosaccharides. However, there are also studies indicating that dectin-1 can collaborate with other TLRs or complement receptor 3 (45, 46). Further investigation into the possible role of dectin-1 might be necessary to rule out any collaboration with other receptors. In conclusion, this *in vitro* study indicates that combined exposure of IECs to CpG ODN and scFOS/lcFOS in a peanut-specific co-culture model contributes to an effector response that is favored toward a Th1 and regulatory IL-10 response and is less prone to the Th2 milieu. To improve efficacy and safety of currently developing protocols for immunotherapy, scFOS/lcFOS may be an interesting candidate for dietary adjunct therapy in allergen-specific immunotherapy, since the final efficacy goal of immunotherapy is the suppression or recovery of the allergen-specific Th2 response which may contribute to acquiring long lasting tolerance induction.

## Ethics Statement

All subjects gave written informed consent in accordance with the Declaration of Helsinki. The protocol was approved by the Ethics Committee of the University Medical Center Utrecht (NL51606.041.15).

## Author Contributions

SH, LW, and HO designed the experiments. AK assisted in recruitment of patients. SH and SO performed the experimental procedures. SH performed data collection and analyses and drafted the manuscript. JG, AK, SO, LW, and HO contributed to data interpretation and critically revised the manuscript.

## Conflict of Interest Statement

JG and SO are employed by Nutricia Research.
